# Unusual complication following laparoscopic sleeve gastrectomy, a case report

**DOI:** 10.1016/j.ijscr.2025.111656

**Published:** 2025-07-10

**Authors:** Awadh Alqahtani

**Affiliations:** Department of Surgery, College of Medicine, King Saud University, King Khalid road, P.O. Box, 7805, Riyadh 11472 DEM 65, Saudi Arabia

**Keywords:** Laparoscopic sleeve gastrectomy, Renal infarction, Venous thromboembolism, Obesity, Postoperative complications, Case report

## Abstract

**Introduction and importance:**

Venous thromboembolic events (VTE) are a rare but significant postoperative complication following bariatric surgery, with an incidence of approximately 1 %. This case report presents a 42-year-old female who developed a renal vein thrombosis leading to renal infarction following laparoscopic sleeve gastrectomy (LSG) which was a non-paradoxical embolism. To our knowledge no similar case reported in the literature.

**Presentation of case:**

A 42-year-old woman with obesity (BMI 35.1) and dyslipidemia underwent LSG with prophylactic anticoagulation. Three days post-op, she presented with sudden left flank pain and gross hematuria. Her laboratories were significant for elevated D-dimer and inflammatory markers. Renal function was preserved. An enhanced imaging revealed left renal infarction (60–70 % hypoperfusion) with no arterial or venous thrombi. She was treated with therapeutic anticoagulation for six months. No underlying structural or hematologic causes were found, so the infarction was likely provoked by surgery, obesity, and dehydration.

**Clinical discussion:**

This case underscores the importance of individualized thromboembolic prophylaxis, adequate postoperative hydration, and vigilant surveillance in high-risk bariatric patients. Given the rarity of renal infarction post-LSG, further studies are warranted to guide preventative strategies and optimize patient outcomes.

**Conclusion:**

This rare case of renal infarction after LSG underscores the need for quick diagnosis and team-based care to ensure the best outcome. Postoperative vigilance—hydration, personalized anticoagulation, and close follow-up—are key in reducing VTE post bariatric surgery.

## Highlights

• Venous thromboembolic events can occur following bariatric surgery in an unusual locations.
• Renal infarction following bariatric surgery can simply present as abdominal pain.
• Management of such complication should include prevention and multidisciplinary team involvement.

## 1 Introduction

Venous thromboembolic events (VTE) as a postoperative complication after bariatric surgery are reported at rate of 1 % [1,2]. Several factors can contribute to such complication in the context of bariatric surgery including patient's characteristics, obesity related comorbidities, and prolonged laparoscopic surgery [3,4]. Most of VTE occurring after LSG are portal vein thrombosis, deep venous thrombosis, and pulmonary embolism [5,6]. In this case report, we mention a rare postoperative complication in the form of a VTE following LSG which involved the left kidney caused by left renal vein thrombosis. This case is reported according to SCARCE guidelines [7].

## 2 Case presentation

A 42-year-old female, with a history of dyslipidemia managed by diet, obesity (BMI 35.1, class II), and prior surgical interventions including cesarean section, septoplasty, and laparoscopic treatment of ectopic pregnancy, presented with a longstanding history of progressive weight gain. Despite multiple attempts at lifestyle modifications and dietary regimens, her efforts to achieve significant weight reduction were unsuccessful.

In light of her clinical profile, she underwent an uneventful LSG. At the operating room, patient positioning, application of pneumatic compression device and administration of antibiotic prophylaxis were done. After anesthesia induction, preparation of surgical filed was completed. Abdomen was entered using 5 mm vesiport superior and to the left of umbilicus. Other ports were 5 mm at left upper quadrant, 15 mm at superior and to the right of umbilicus, 5 mm port at right upper quadrant, and a Nathanson liver retractor at epigastric area. The greater omentum was divided till the gastroesophageal junction, followed by applying 60 mm black Ethicon Tristapler at the antrum followed by purple staplers along a 36Fr bougie. Then clips are applied along the staple line followed by staple line reinforcement with 3–0 suture.

As per guideline she was covered by prophylactic anticoagulation before & after surgery while inpatient. Patients are encouraged to ambulate and use the incentive spirometry after the surgery. An established protocol is followed regarding pain medications, anti-coagulants, and progression of oral intake. She was discharged the following day in stable condition with no immediate postoperative complications with a prophylactic dose of anticoagulation for 21 days duration.

**Fig. 1:**
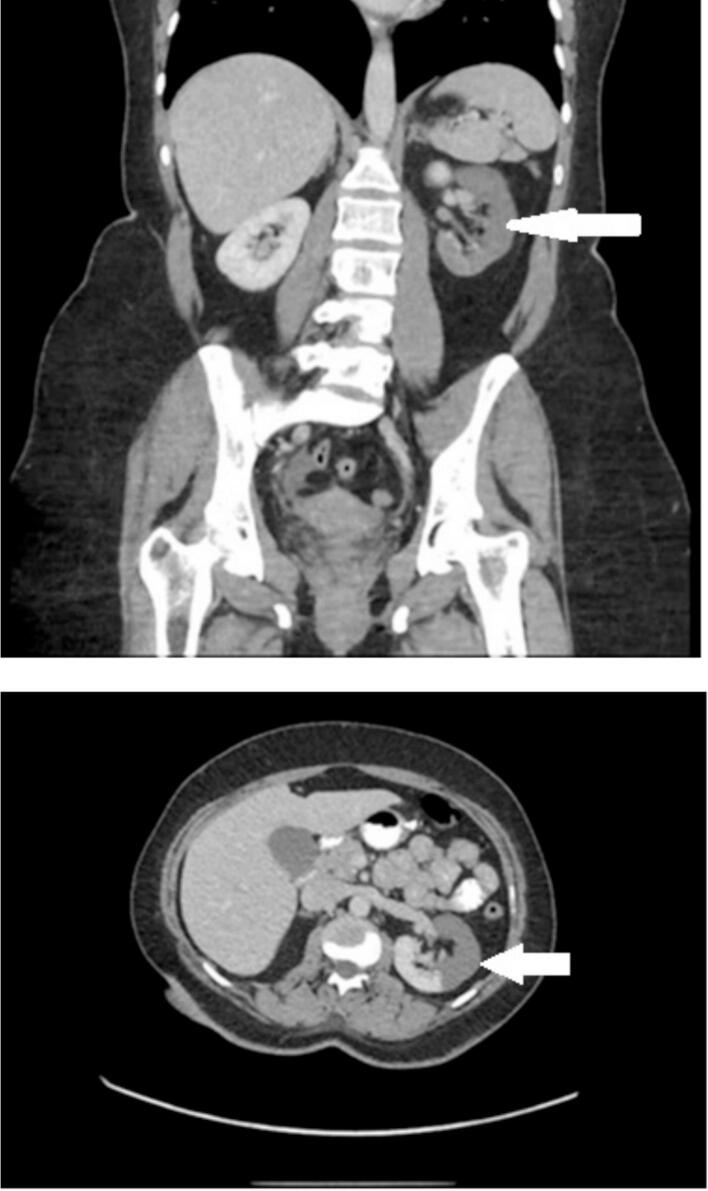
An enhanced CT scan of the patient showing hypoperfusion of the left kidney, affecting approximately 60–70 % of the organ with sparing of the posterior medial region. Renal arteries and vein were patent.

On postoperative day three, the patient came to the Emergency Department with complain of having severe sudden-onset, left flank pain. Her vitals upon arrival was stable beside borderline tachycardia. The physical examination was significant for dehydration, distress, with no abdominal peritonitis. The examination however was positive for left flank tenderness. Initial blood workup including showed elevation of white blood cells, C-reactive protein, D-dimer, and elevated. Renal function and coagulation profile were within normal limits. Urine Analysis came strong positive for gross blood. A triphasic contrast-enhanced computed tomography of the abdomen and pelvis revealed hypoperfusion of the left kidney, affecting approximately 60–70 % of the organ with sparing of the posterior medial region (Fig. 1). The renal arteries and vein were clear from any thrombosis probably because the patient was on prophylactic anticoagulation. An ultrasonography with doppler of the left kidney showed hypoechoic cortical area, with no evidence of stenosis or affected blood flow in the left renal arteries and veins. An echocardiogram was done and showed no abnormal pathology rolling out possibility of paradoxical arterial embolus. The diagnosis was made by exclusion and was consistent with renal vein thrombosis.

The patient was admitted for further evaluation and management. Hematology workup including morphology of all blood components, protein S and C levels, antithrombin III activity/antigen, antilupus anticoagulants, and anticardiolipin all came within normal limits. Therapeutic anticoagulation with low molecular weight heparin (LMWH) was initiated. Comprehensive evaluations by cardiology, vascular surgery, and nephrology excluded structural or functional abnormalities contributing to the infarction. Subsequent hematology consultation led to a transition from LMWH to a direct oral anticoagulant (DOAC) for six months. Follow up appointment with bariatric surgery clinic showed a BMI of 26, normal renal function, and normal D-dimer. The patient was satisfied with the management and the outcome. A visit to the hematology recommended to hold the anticoagulation.

## 3 Discussion

Renal infarction following LSG is an extremely rare complication. Its occurrence may be attributed to several contributing factors [6]:

Hypercoagulable State:

Obesity and the postoperative period increase thrombotic risk.

Surgical stress, coupled with postoperative immobility, further exacerbates this condition.

Dehydration:

Reduced oral intake and potential hypovolemia following LSG predispose to renal hypoperfusion or thrombosis.

Renal vein thrombosis:

Thromboembolic events may occur in patients with undiagnosed hypercoagulable disorders or vascular anomalies.

Embolic phenomena:

Emboli may result from cardiac or aortic abnormalities exacerbated by perioperative stress.

In addition, Renal infarction typically manifests with: Acute flank pain commonly unilateral, Microscopic or gross hematuria, Inflammatory responses such as fever (less common), and lastly Hypertension due to activation of the renin-angiotensin system [8].

In the context of bariatric surgery, patients who are subjected to bariatric surgery are complex in their medical conditions and have a significant risk for VTE in addition to the excess weight. These type of patients are commonly diagnosed with obesity associated conditions, possibly practicing habits like smoking, in addition to the duration of the procedure that might be prolonged which all collectively increase the risk of VTE [9]. Hence the American Society of Metabolic and Bariatric Surgery recommends VTE prophylaxis including mechanical prophylaxis and chemoprophylaxis during admission, in addition to extended prophylaxis for 2 to 3 weeks after discharge [10].

In this case, the diagnosis of renal infarction was confirmed through was confirmed through a contrast-enhanced CT which identified wedge-shaped perfusion defects & by laboratory investigations which showed elevated lactate dehydrogenase (LDH) and D-dimer levels. Normal serum creatinine indicated preserved renal function due to subsegmental involvement. Although there were no thrombus found on imaging, and considering that the patient is at a relatively high risk for thrombosis (obesity, laparoscopic surgery, postoperative period with risk of dehydration) a compelling hypothesis is that the patient developed thrombus but it go dissolved because she was on anticoagulation and rehydrated which made the thrombus dissolve before conforming it with an image.

The patient's condition was managed with therapeutic anticoagulation, initially with low molecular-weight heparin (enoxaparin), later transitioned to direct oral anticoagulant therapy (apixaban). Adequate hydration and close monitoring of renal function were ensured throughout the course of treatment.

To mitigate the risk of similar complications, the following measures are recommended which includes: (1) hydration optimization by ensuring sufficient postoperative fluid intake. (2) Risk-adjusted anticoagulation tailored to individual thrombotic risk profiles. (3) Postoperative surveillance by monitoring of renal function and coagulation parameters in high-risk patients.

Upon reviewing the literature, we found only one case reporting a renal infarction. This case was significant for a patent foramen ovale which explains a possible paradoxical embolus causing arterial infarction [6]. In the workup of our case, there were no cardiac anomalies that could precipitate to an arterial embolus which was not reported before.

## 4 Conclusion

This case highlights an exceptionally rare complication of LSG and emphasizes the critical importance of prompt diagnosis and interdisciplinary management involving hematology, nephrology, cardiology, and vascular surgery specialists in ensuring successful outcomes in complex clinical scenarios. A vigilant postoperative approach, including optimization of hydration, tailored anticoagulation, and regular follow-up, is essential for minimizing the risk of thromboembolic complications in bariatric surgery patients.

## CRediT authorship contribution statement

Dr. Awadh Alqahtani: writing of paper, review of literature, collecting images.

## Consent

Taken from the patient.

## Ethical approval

Exempted.

## Guarantor

Dr. Awadh Alqahtani.

## Declaration of Generative AI and AI-assisted technologies in the writing process

No artificial intelligence was utilized for this manuscript.

## Sources of funding

None.

## Declaration of competing interest

None.

## References

[1] Dang J.T., Switzer N., Delisle M., Laffin M., Gill R., Birch D.W., Karmali S. (2019 Mar 15). Predicting venous thromboembolism following laparoscopic bariatric surgery: development of the BariClot tool using the MBSAQIP database. Surg. Endosc..

[2] El Chaar M., Alvarado L., Nimeri A., Dey T., Clapp B., Rogers A.M., Petrick A.T. (2024 Oct 15). Development of venous thromboembolic event risk calculator for metabolic and bariatric surgery patients to reduce mortality. Surg. Obes. Relat. Dis..

[3] Darvall K.A., Sam R.C., Silverman S.H., Bradbury A.W., Adam D.J. (2007 Feb 1). Obesity and thrombosis. Eur. J. Vasc. Endovasc. Surg..

[4] Chan M.M., Hamza N., Ammori B.J. (2013 Jan 1). Duration of surgery independently influences risk of venous thromboembolism after laparoscopic bariatric surgery. Surg. Obes. Relat. Dis..

[5] Giannis D., Geropoulos G., Kakos C.D., Lu W., El Hadwe S., Fornasiero M., Robertson A., Parmar C. (2023 Oct). Portomesenteric vein thrombosis in patients undergoing sleeve gastrectomy: an updated systematic review and meta-analysis of 101,914 patients. Obes. Surg..

[6] Khoma O., Suppiah A., Martin D. (2016 Jan 1). Case report: renal infarction by paradoxical embolism through the patent foramen ovale as an unusual cause of post-operative abdominal pain after sleeve gastrectomy. Int. J. Surg. Case Rep..

[7] Kerwan A., Al-Jabir A., Mathew G., Sohrabi C., Rashid R., Franchi T., Nicola M., Agha M., Agha R.A. (2025). Revised surgical CAse REport (SCARE) guideline: an update for the age of artificial intelligence. Premier Journal of Science.

[8] Gambhir S., Inaba C.S., Alizadeh R.F., Nahmias J., Hinojosa M., Smith B.R., Nguyen N.T., Daly S. (2020 Aug). Venous thromboembolism risk for the contemporary bariatric surgeon. Surg. Endosc..

[9] Finks J.F., English W.J., Carlin A.M., Krause K.R., Share D.A., Banerjee M., Birkmeyer J.D., Birkmeyer N.J., Michigan Bariatric Surgery Collaborative (2012 Jun 1). Predicting risk for venous thromboembolism with bariatric surgery: results from the Michigan Bariatric Surgery Collaborative. Ann. Surg..

[10] Bariatric Surgery Clinical Issues Committee (2013). ASMBS updated position statement on prophylactic measures to reduce the risk of venous thromboembolism in bariatric surgery patients. Surgery for obesity and related diseases: official journal of the American Society for Bariatric Surgery..

